# Liver volume-based prediction model stratifies risks for hepatocellular carcinoma in chronic hepatitis B patients on surveillance

**DOI:** 10.1371/journal.pone.0190261

**Published:** 2018-01-02

**Authors:** Chung Seop Lee, Yong Jin Jung, Soon Sun Kim, Jae Youn Cheong, Ga Ram Lee, Han Gyeol Kim, Beom Hee Kim, Jung Wha Chung, Eun Sun Jang, Sook-Hyang Jeong, Kyung Ho Lee, Jin-Wook Kim

**Affiliations:** 1 Department of Medicine, Seoul National University Bundang Hospital, Seongnam, Republic of Korea; 2 Department of Internal Medicine, Seoul National University College of Medicine, Seoul, Republic of Korea; 3 Department of Internal Medicine, SMG-SNU Boramae Medical Center, Seoul, Republic of Korea; 4 Department of Gastroenterology, Ajou University School of Medicine, Suwon, Republic of Korea; 5 Department of Radiology, Seoul National University College of Medicine, Seoul, Republic of Korea; Universita degli Studi di Pisa, ITALY

## Abstract

**Background and aim:**

The aim of this study was to determine whether dynamic computed tomography (CT)-measured liver volume predicts the risk of hepatocellular carcinoma (HCC) when the CT scans do not reveal evidence of HCC in chronic hepatitis B (CHB) patients on surveillance.

**Methods:**

This retrospective multicentre cohort study included 1,246 patients who received entecavir and regular HCC surveillance in three tertiary referral centres in South Korea. Liver volumes were measured on portal venous phase CT images. A nomogram was developed based on Cox independent predictors and externally validated. Time-dependent receiver operating characteristic (ROC) analysis was performed for comparison with previous prediction models.

**Results:**

Patients who received dynamic CT studies during surveillance had significantly higher risk for HCC compared to patients without CT studies (hazard ratio [HR] = 3.1; p < 0.001). Expected/measured liver volume ratio was an independent predictor of HCC (HR = 4.2; p = 0.002) in addition to age, sex and cirrhosis. The nomogram based on the four predictors discriminated risks for HCC (HR = 4.1 and 6.0 in derivation and validation cohort, respectively, for volume score > 150; p < 0.001). Time-dependent ROC analysis confirmed better performance of the volume score compared to HCC prediction models with conventional predictors (integrated area under curve = 0.758 vs. 0.661–0.712; p < 0.05).

**Conclusions:**

CT-measured liver volume is an independent predictor of future HCC, and nomogram-based liver volume score may stratify the risks of HCC in CHB patients who showed negative CT findings for HCC during surveillance.

## Introduction

Chronic hepatitis B virus (HBV) infection is the leading cause of HCC worldwide [1] and surveillance is recommended for CHB patients with increased risk for HCC [2–5]. Ultrasound (US) examination is the main screening tool for HCC surveillance, but dynamic imaging techniques such as 4-phase multidetector computed tomography (CT) or dynamic contrast enhancement magnetic resonance imaging (MRI) are requested for the diagnostic confirmation if US detects suspicious lesions [2, 3, 5–7]. Dynamic imaging studies are also considered during surveillance when tumour marker levels increase unexpectedly or poor sonic window prevents adequate US assessment [3, 5, 8]. If dynamic imaging does not reveal definite evidence of HCC, either biopsy or close follow-up is recommended [2, 3, 5, 6]. However, biopsy has not only risks but also limitations in distinguishing well-differentiated HCC from other non-malignant macronodules [9]. Therefore, “enhanced follow-up” [2] is frequently opted for according to the stratified risk for HCC when dynamic imaging studies do not reveal diagnostic findings of HCC. However, the clinical outcome of this patient group has not been well-defined, even less risk stratification strategies.

Liver volume decreases as hepatic fibrosis progresses in CHB [10, 11]. Liver volume correlates with degree of hepatic dysfunction in liver cirrhosis [12, 13], but it may decrease even when liver function is still well preserved.[12, 13]. Liver volumetry has been successfully used for preoperative planning for major hepatic resections and living related donor liver transplantation [14, 15]. It can be speculated that small liver size may represent increased risk for HCC as a consequence of prolonged hepatic fibrosis, but this intuitive notion has not yet been quantitatively elucidated in chronic viral hepatitis.

In this study, we sought to determine whether CT-measured liver volume predicts future development of HCC when the CT scans during surveillance do not show evidence of HCC in chronic hepatitis B (CHB) patients.

## Materials and methods

### Patients

This multicentre retrospective cohort study included consecutive CHB patients who started entecavir (ETV) therapy, one of the first-line oral antiviral agent for chronic HBV infection, between 2007 and 2016 in three tertiary care centres. The institutional review board (IRB) and ethnics committee of each participating hospital approved this study (Seoul National University Bundang Hospital IRB no. B-1609/361-102, and Ajou University Hospital IRB no. AJIRB-BMR-KSP-13-168). Clinical investigations were conducted according to the principles expressed in the Declaration of Helsinki. The requirement for informed consent was waived by the IRBs due to the retrospective nature of the study and the anonymous analysis of the data.

The inclusion criteria were CHB patients over 18 years who received ETV as an initial antiviral therapy. Patients were excluded if any of the following criteria was met: 1) duration of HCC surveillance < 12 months; 2) diagnosis of HCC before or within 6 months after the initial screening; 3) Child-Pugh class C cirrhosis; or 4) HCV or HIV coinfection, other malignancies or organ transplantation. The index date was set to the date of initiation of ETV.

**Fig 1:**
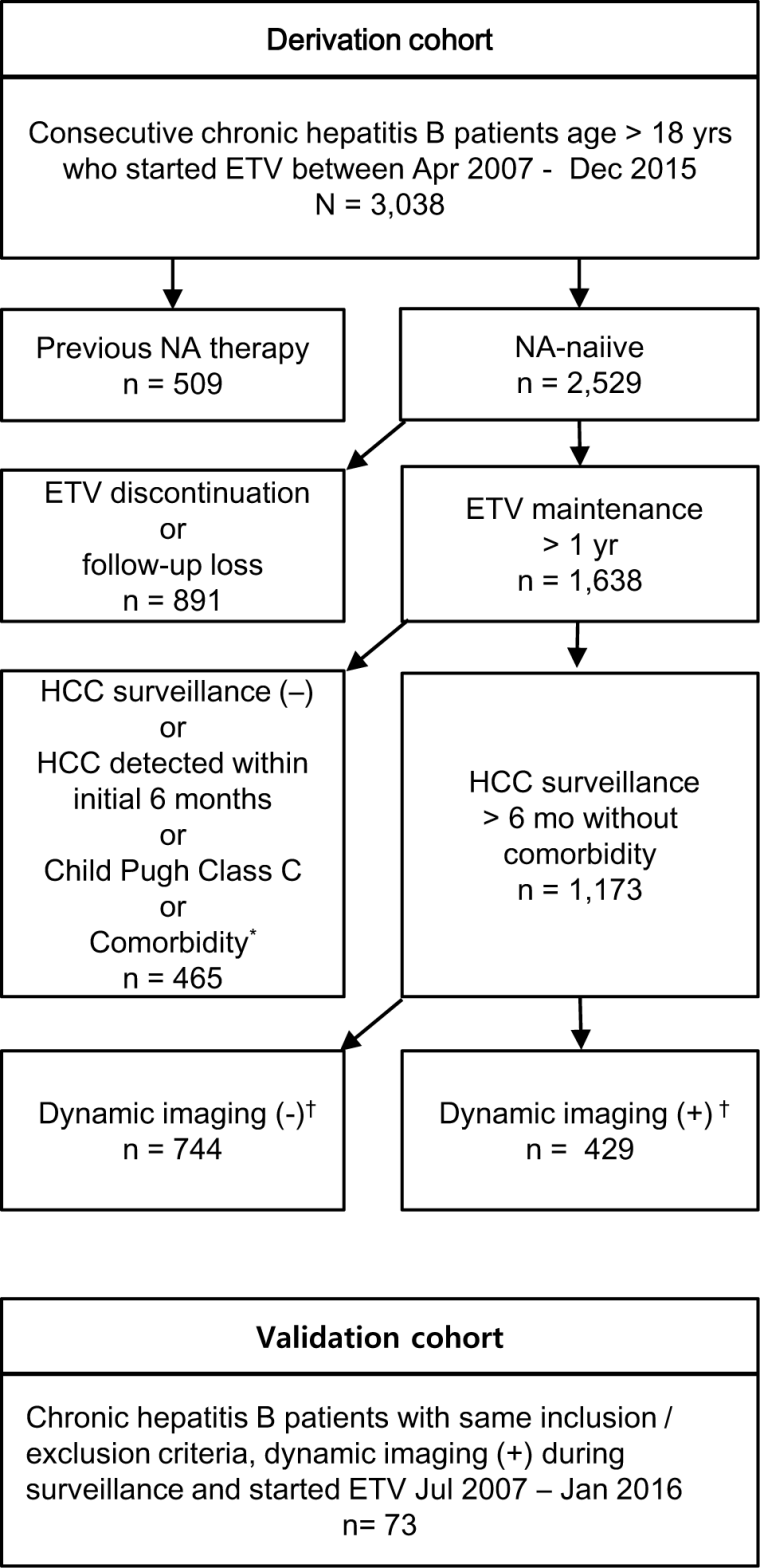
Flow chart of the study population. *HCV or HIV coinfection, other malignancy or organ transplantation. ^†^Dynamic imaging (+) subgroup received at least one dynamic CT study during surveillance which revealed no evidence of HCC: this subgroup served as the derivation dataset for liver volume analysis. No dynamic imaging subgroup did not receive dynamic CT studies during surveillance, except for the confirmative imaging tests in case of HCC. ETV, entecavir; HCC, hepatocellular carcinoma; NA, nucleos(t)ide analogue.

The study population was composed of derivation and validation cohort (Fig 1). The derivation cohort (n = 1,173) was built from consecutive patients in the previously reported liver disease registry [16]. Among the patients in the derivation cohort, 429 received at least one multidetector CT scan which did not reveal HCC and the other 744 did not receive CT scans except for the confirmative CT in case of HCC detection (Table 1). The validation cohort was composed of 73 consecutive patients from two separate centres who met the same inclusion / exclusion criteria and received at least one multidetector CT scan during surveillance.

**Table 1:** Characteristics of the study population.

Patients group	Derivation cohort		P value	Validation cohort	P value*
	(single centre, n = 1,173)			(two centres, n = 73)	
	Dynamic CT-	Dynamic CT+		Dynamic CT+	
	(n = 744)	(n = 429)		(n = 73)	
Duration of follow-up (Mo) Age, years	50 (49) 46 (15)	47 (67) 51 (15)	0.535 < 0.001	43 (45) 56 (15)	0.492 < 0.001
Male, n (%)	445 (60)	274 (64)	0.171	43 (60)	0.432
Liver cirrhosis, n (%)	222 (30)	278 (65)	< 0.001	49 (67)	0.692
Alpha-fetoprotein (ng/mL)	3.6 (4.1)	6.2 (19.6)	< 0.001	8.6 (19.0)	0.989
Albumin (mg/dl)	4.3 (0.7)	4.1 (0.7)	< 0.001	4.0 (0.7)	0.250
Bilirubin (mg/dl)	0.9 (0.5)	1.1 (0.7)	< 0.001	1. 1 (0.6)	0.203
HBV DNA (Log IU/L)	5.88 (3.59)	5.23 (4.40)	< 0.001	6.38 (1.77)	< 0.001
HBeAg positivity, n (%)	375 (51)	192 (46)	0.058	29 (40)	0.375
ALT (IU/L)	93 (123)	53 (69)	0.510	51 (47)	0.093
Platelet count, ×10^9^/L	178 (75)	140 (82)	< 0.001	133 (67)	0.505
Prothrombin time (INR)	1.06 (0.10)	1.12 (0.20)	< 0.001	1.13 (0.38)	0.818
CT-measured liver volume (mL)	-	1138 (404)	-	1043 (375)	0.065
Volume index†	-	0.96 (0.27)		0.97 (0.25)	0.411

*between derivation and validation cohort.

†Volume Index = $\frac{Formula\;liver\;volume}{CT-measured\;liver\;volume}$

Continuous variables are expressed as the median (interquartile range). P values are calculated by t-test or χ^2^ test for continuous and categorical variables, respectively. HBeAg, hepatitis B e-antigen; ALT, alanine aminotransferase.

All patients were evaluated with biochemical and virologic blood tests before ETV treatment and at 3- to 6-month intervals thereafter. The surveillance program consisted of both abdominal US and serum alpha-fetoprotein at 6-month intervals. Dynamic imaging studies, i.e., 4-phase multidetector CT or dynamic contrast enhanced MRI, were performed if US showed > 1 cm new nodule(s) or if serially measured AFP levels increased progressively. Dynamic imaging was also considered at the discretion of the attending physicians when US examination was considered inadequate for detection of possible small HCC [5, 6, 8, 17]. Diverse models of ultrasonography, CT, and MR machines from different manufacturers were used during the 10-year study period in the three investigating sites.

Liver cirrhosis was diagnosed histologically or clinically. Clinical liver cirrhosis was defined as US features of cirrhosis (coarse liver echotexture with nodularity) plus evidence of portal hypertension including ascites, splenomegaly, thrombocytopenia (< 100x10^9^/L) and varices [18]. HCC was diagnosed by biopsy or typical enhancing patterns on dynamic imaging techniques [5, 19].

### Measurement of liver volume

Liver volume was measured on portal venous phase contrast-enhanced CT images which were obtained within 6 months from the index date. The entire sections of portal phase liver images were downloaded, transformed to a stack and outlined for calculation of cross-sectional area by using ImageJ version 1.50i (http://imagej.nih.gov/ij) [20]. To reduce measurement errors, the liver boundaries were semi-automatically determined using the Versatile Wand Tool (https://imagej.nih.gov/ij/plugins/versatile-wand-tool/index.html, S1 Video).The inferior vena cava and gallbladder were excluded from selection, whereas the intrahepatic portal veins were included in the measured areas. The automatically calculated area of the liver was summed and multiplied by the image interval. In order to correct the effect of body build, liver volume index was calculated as a standard-to-measured volume ratio [13]:

$$\mathrm{Liver}\;\mathrm{volume}\;\mathrm{index}=\frac{Formula\;liver\;volume\;(ml)}{CT-measured\;liver\;volume\;(ml)}$$

where the formula liver volume was deduced from the body surface area (BSA): *Formula liver volume(ml) = 893*.*485 × BSA—439*.*169 (mL)* [21]. The BSA was estimated with Du Bois’ formula: *BSA = 0*.*007184 × (weight in kg)* ^*0*.*425*^ *× (height in cm)*^*0*.*725*^ [22].

Liver volume was measured by single investigator (CSL). To assess the reproducibility of volumetry, thirty patients were randomly selected and three different medical students measured live volume of the same patient. The inter-observer variability of volumetry was assessed by Bland-Altman plot analysis [23].

### Liver volume based HCC prediction model

Cox proportional hazard analysis was used to identify the independent predictors of future HCC development. Next, we generated a nomogram from these independent predictors by using R package *rms* in order to estimate the HCC risks in CHB. The performance of the HCC prediction nomogram was tested by predictiveness curves, calibration and discrimination analyses as recommended by the *Transparent reporting of a multivariable prediction model for individual prognosis or diagnosis* (TRIPOD) statement [24]. Predictiveness curve was drawn by R package WPC in order to visualize the impact of the volume score nomogram in HCC prediction [25]. Calibration curves were plotted using the R package *rms* with 150 bootstrap iterations. Kaplan-Meier curves were plotted for the discrimination analysis with the log-rank test.

The performances of the HCC prediction models were compared with previous prediction models by time-dependent receiver operating characteristic (ROC) analyses of the Cox model [26]. The integrated areas under the curves (iAUCs) were calculated with the R package *risksetROC* with 100 bootstrap iterations. The obtained iAUC was compared between the nomogram-based prediction model and the representative HCC risk prediction models, i.e., GAG-HCC score (age, sex, HBV DNA and cirrhosis)[27], CU-HCC score (age, albumin, bilirubin, HBV DNA and liver cirrhosis) [28] and PAGE-B score (age, sex and platelet counts) [29].

### Statistical analysis

The statistical analyses were performed using STATA 14.2 (Stata Corp LLC, TX, USA) and R statistical package version 3.3.1. Continuous and categorical variables were analysed with t-tests and chi-square tests, respectively. A p value of <0.05 was considered significant.

## Results

### Baseline characteristics and HCC incidence of study cohorts

**Fig 2:**
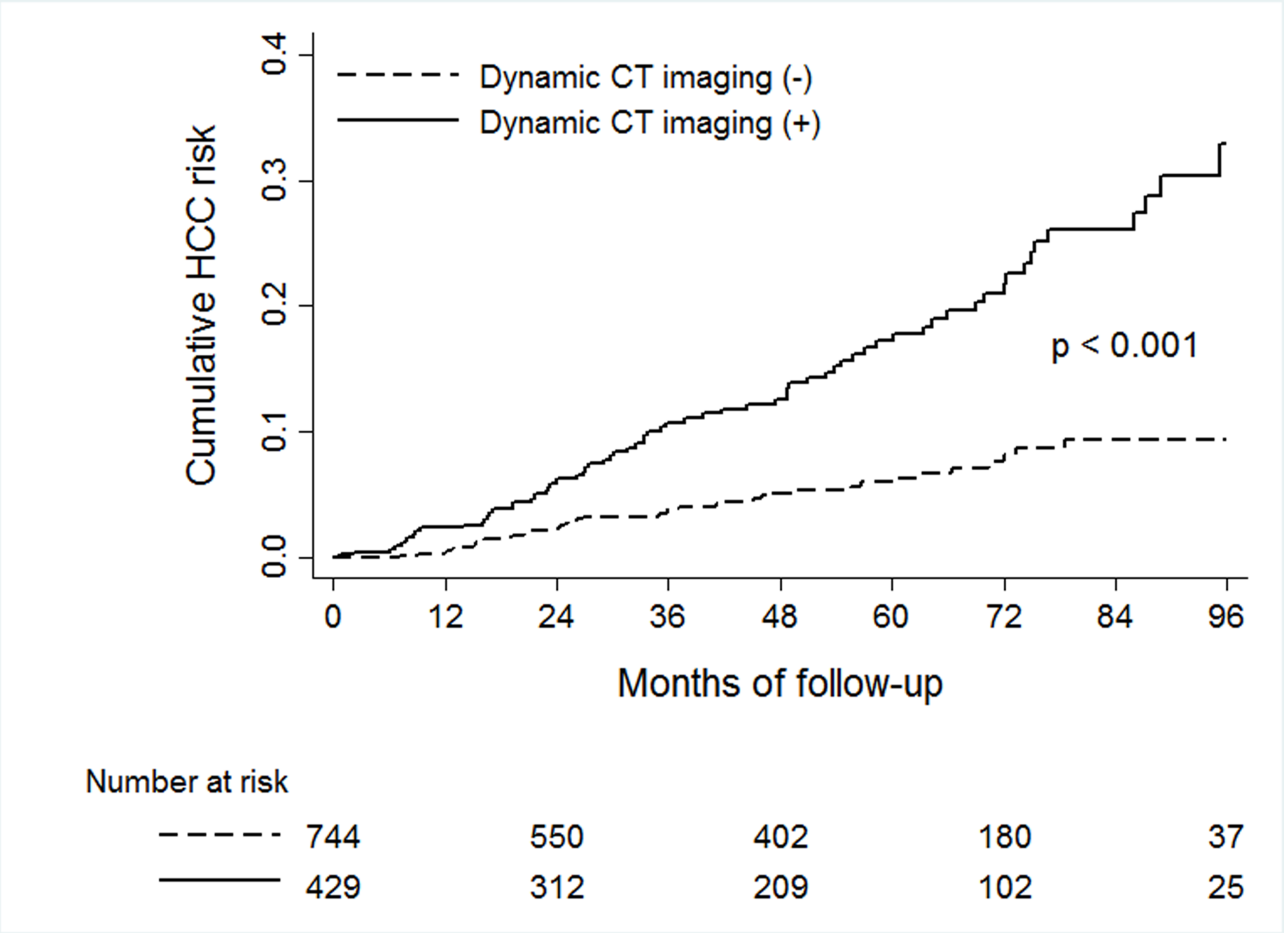
Kaplan-Meier analysis of HCC incidence in the derivation cohort.

The baseline characteristics of the study cohorts are presented in Table 1. Analysis of the derivation cohort revealed that patients who received dynamic CT (n = 429) showed older age, higher frequency of live cirrhosis, higher AFP levels and more advanced liver disease compared to patients without dynamic CT (n = 744). These results suggested that patients with increased risk for HCC were more likely to received dynamic CT studies during surveillance. Kaplan-Meier analysis confirmed that the incidence rate of HCC was significantly higher in patients with dynamic CT imaging compared to patients without CT imaging (3.3 vs. 1.1 per 1000 person-year, respectively; p < 0.001 by log-rank test) (Fig 2). Cox univariate analysis also indicated that patients with dynamic CT imaging had significantly increased risk for HCC (hazard ratio [HR] = 3.1, 95% CI = 1.1–4.6; p < 0.001). There were no significant differences between the derivation and validation cohort except for older age and higher baseline HBV DNA loads in the validation cohort.

### Liver volume as an independent predictor of HCC

Next, we sought to determine whether CT-measured liver volume predicts future development of HCC. Bland-Altman plot showed that the reproducibility of volumetry was good with limits of agreement ranging between -7.4% and 5.4% (S1 Fig). Univariate Cox analysis of the 429 patients revealed that old age, male sex, presence of cirrhosis, low platelet count, detection of hypovascular nodules(s) by dynamic imaging techniques and high liver volume index were predictors of HCC development (Table 2). Multivariate analysis confirmed that decreased liver volume, indicated by high liver volume index, was an independent risk factor for HCC, along with old age, male sex and presence of cirrhosis. Sensitivity analysis showed that the volume index remained significant among patients with cirrhosis (HR = 5.1; 95% CI = 2.1–12.3; p < 0.001).

**Table 2:** Predictors of HCC development by Cox proportional hazard model.

	Univariate			Multivariate		
Parameters	HR	95% CI	P value	HR	95% CI	P value
Age	1.04	1.02–1.06	**0.001**	1.03	1.01–1.06	**0.013**
Male sex	1.86	1.05–3.29	**0.034**	2.00	1.12–3.56	**0.020**
Cirrhosis	5.04	2.18–11.65	**< 0.001**	3.05	1.23–7.55	**0.016**
HBV DNA (log IU/mL)	0.97	0.86–1.08	0.546			
HBeAg positivity	0.76	0.47–1.23	0.266			
AFP (ng/mL)	1.00	0.99–1.00	0.067			
Albumin (g/dL)	0.71	0.50–1.01	0.057			
Bilirubin (mg/dL)	0.98	0.87–1.11	0.606			
PT INR	1.68	0.73–3.81	0.220			
Platelet	0.99	0.98–0.99	**< 0.001**	0.99	0.99–1.00	0.089
Hypovascular nodule(s)	1.76	1.04–2.99	**0.035**	1.12	0.65–1.92	0.686
Volume Index*	7.81	3.37–18.09	**< 0.001**	4.23	1.72–10.40	**0.002**

Abbreviation: AFP, alpha-fetoprotein; CI, confidence interval; HR, hazard ratio. P values are calculated by Cox regression analysis.

*Volume Index = $\frac{Formula\;liver\;volume}{CT-measured\;liver\;volume}$

Patients with high estimated risk might be not only more likely to receive CT studies but also likely to visit hospitals more frequently, leading to increased detection of HCC. Indeed, the frequency of hospital visits was significantly associated with risk for HCC. However, adjustment for hospital visit frequencies did not significantly change the results of multivariate Cox analysis (S1 Table).

### Development of a liver-volume based model for predicting HCC risk

**Fig 3:**
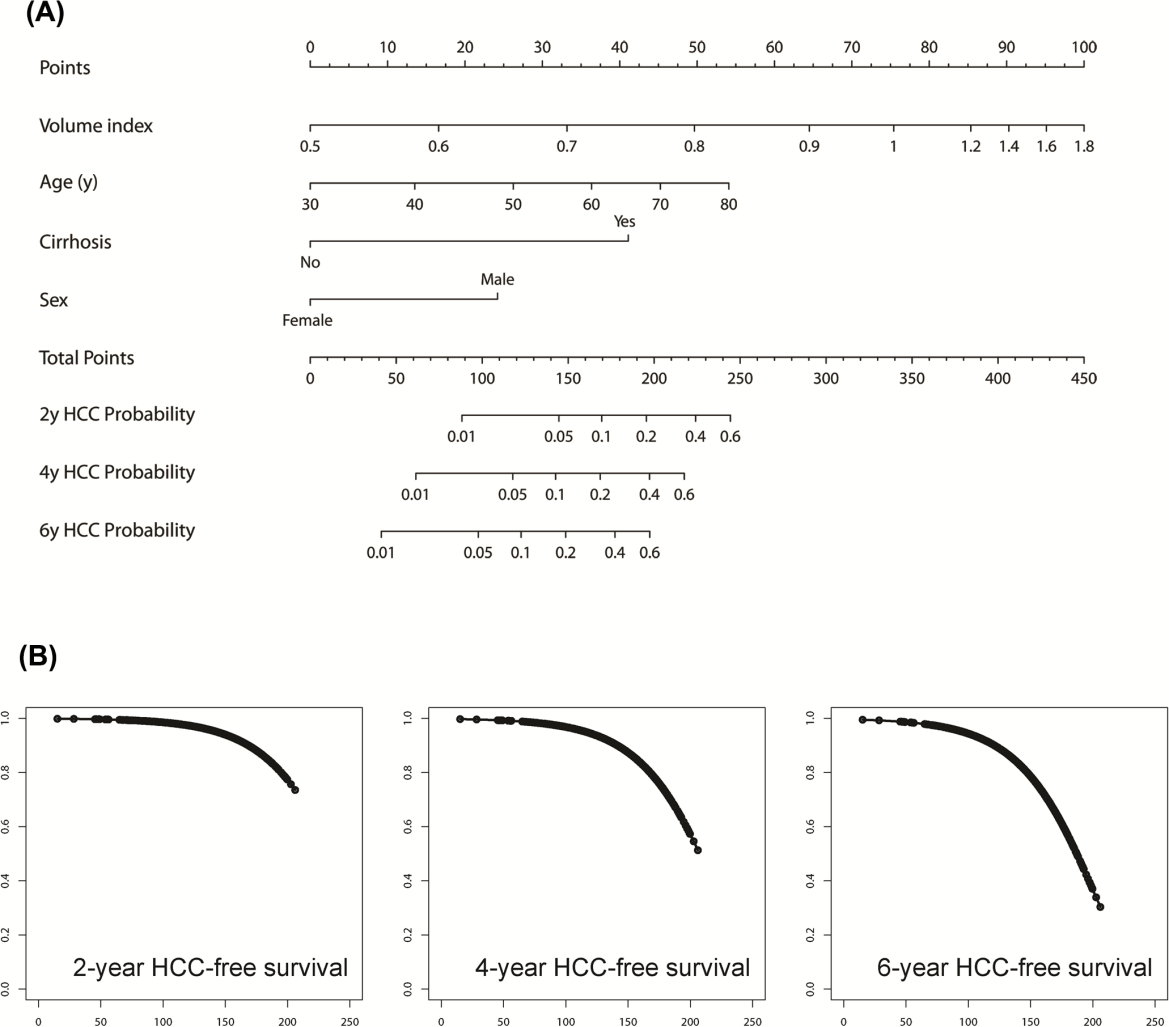
Development of HCC prediction model by Cox analysis. (A) Nomogram for predicting future HCC development based on Cox independent predictors. Points of each parameters (Points axis) are summed to get the total points (Total Points axis), which are then transformed to get the corresponding 2, 4 and 6-yr predicted probability for HCC. (B) Predictiveness curves of nomogram-based volume score (x-axis) plotted against predicted probability of HCC-free survival (Y-axis).

Since liver volume was an independent predictor of HCC, we wanted to develop a liver volume-based model for the prediction of HCC probability. For this purpose, a nomogram was generated based on the Cox independent predictors (Fig 3A). The risk distribution of the score sum of the nomogram, designated as the *volume score*, is presented in Fig 3B. Calibration analysis of the volume score showed fair agreements between the observed and nomogram-predicted HCC probability at 2, 4 and 6-years without significant deviation (S2 Fig).

### Stratification of HCC risk by liver-volume based nomogram

**Fig 4:**
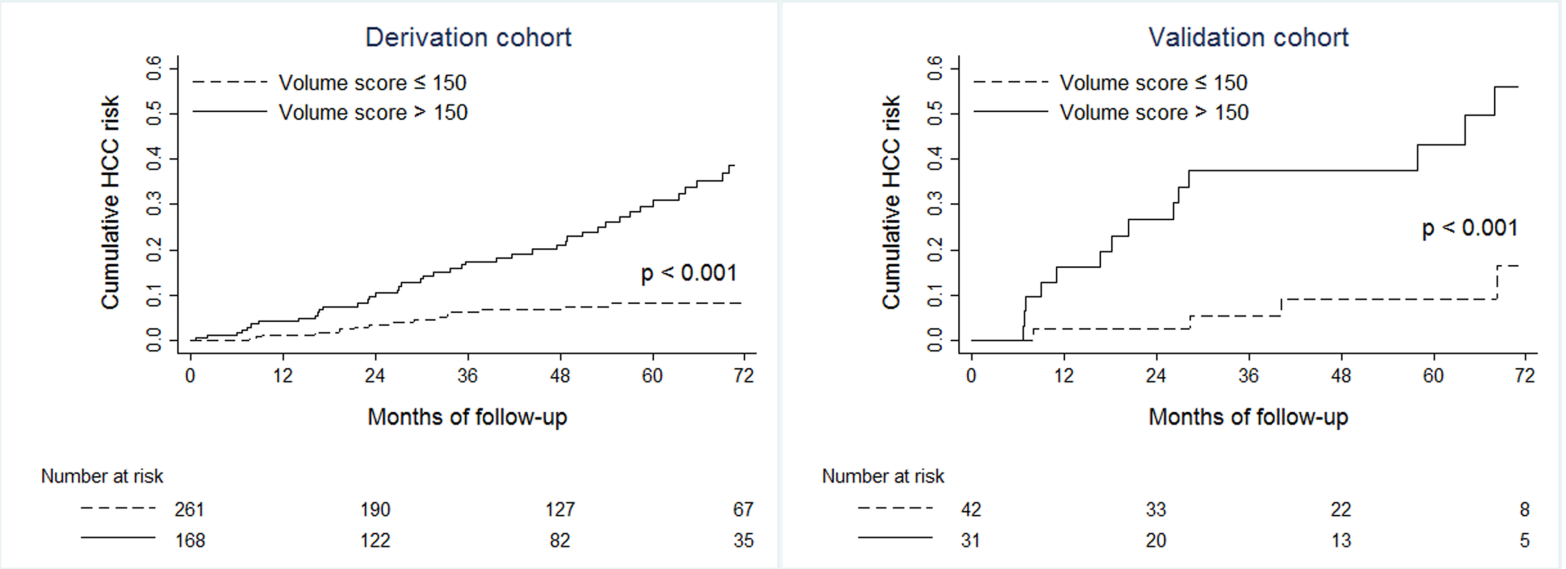
Stratification of HCC probability by nomogram-based liver volume score. Kaplan-Meier probabilities of HCC incidence were plotted according to the liver volume score cut-off of 150 in the derivation and validation cohort.

Next, the nomogram was tested for discrimination in order to determine whether the volume score can stratify the risks of HCC. Predictiveness curves suggested that volume score greater than 150 was associated with a steep increase in the HCC risk. As expected, Kaplan-Meier analysis showed that CHB patients with volume score > 150 had significantly higher risk for HCC in both derivation and validation cohorts (Fig 4). The HR of volume score > 150 was 4.1 (95% CI 2.5–6.9, p < 0.001) and 6.0 (95% CI 2.0–18.0, p < 0.001) in the derivation and validation cohort, respectively.

**Fig 5:**
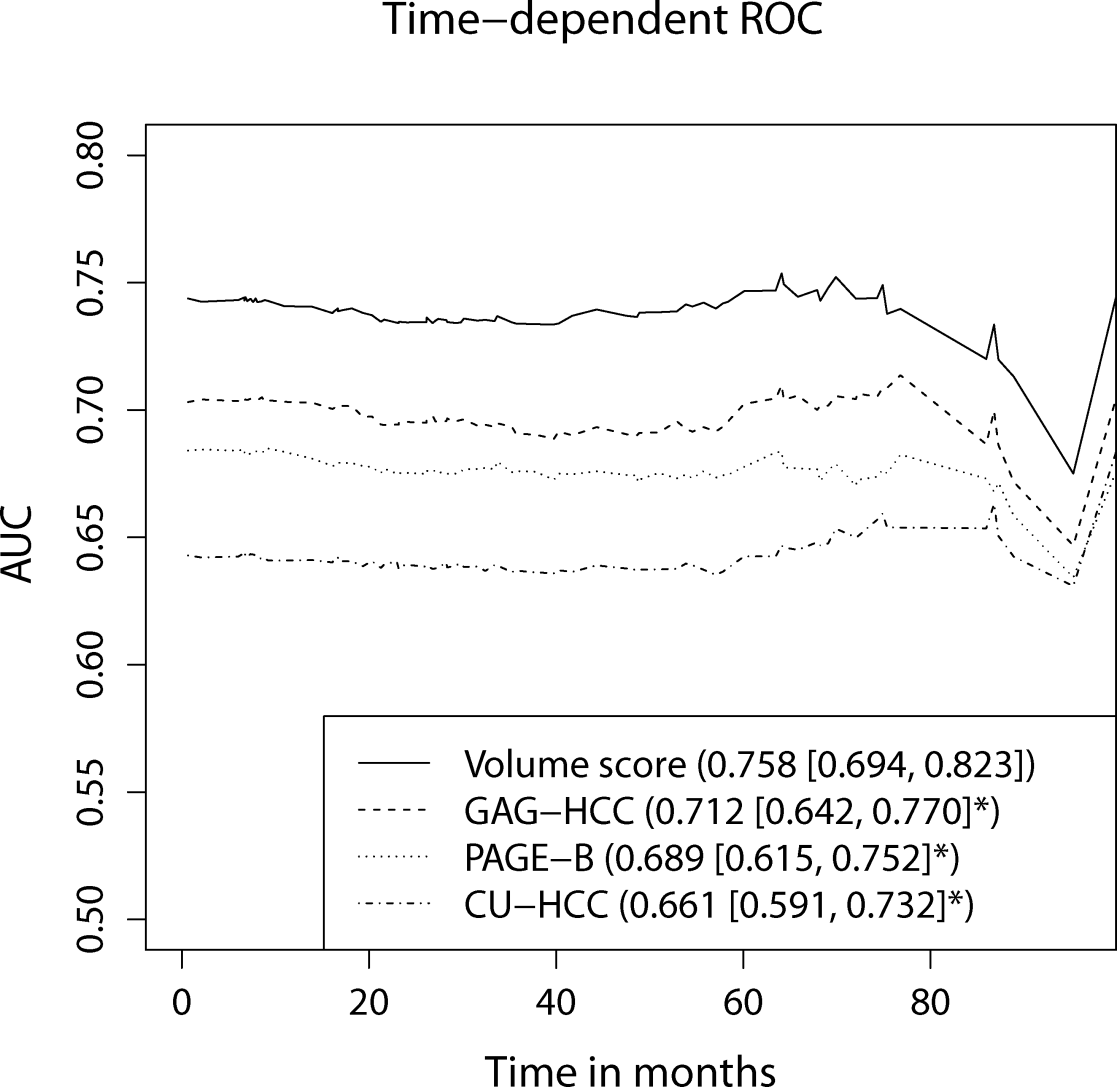
Comparison of HCC probability prediction models by time-dependent ROC analyses. The nomogram-based liver volume score model was compared to previously reported three HCC prediction models. The area under ROC curves (AUCs) were plotted over time for each prediction models. The numbers indicate the integrated AUCs after 100 bootstrapping iterations [95% confidence interval]. Asterisk indicates p < 0.05 against nomogram-based liver volume score model.

Furthermore, time-dependent ROC analysis revealed that the performance of the volume-based prediction model was better compared to the previous HCC prediction models with conventional predictors, i.e., GAG-HCC score [27], CU-HCC score [28] and PAGE-B score [29] with the highest integrated AUC value for the volume-based prediction model (Fig 5, p < 0.05).

## Discussion

Dynamic liver imaging studies are frequently indicated during HCC surveillance in CHB, either as confirmatory tools or as supplementary measures [5, 8] due to suboptimal sensitivity of US in cirrhotic patients with nodular liver parenchyma [30, 31]. Our cohort data showed that about one-thirds (429/1173) of CHB patients on ETV therapy and regular surveillance received at least one dynamic CT scan, which did not reveal definite HCC. We also found that these patients harbour 3-fold increased risk for HCC compared to patients without dynamic CT imaging studies during surveillance. These data may be regarded as rationale for enhanced follow-up in these patients, but HCC risk stratification within this high-risk group has not been elucidated yet.

Our study showed that liver volume index was an independent predictor of HCC development in addition to age, sex and cirrhosis in CHB patients on oral antiviral therapy. Liver cirrhosis is a well-established risk factor for HCC and one of the most influential components of several risk prediction models [27–29, 32]. However, current laboratory parameters such as Child-Turcotte-Pugh score have limitations in further stratifying the HCC risks in compensated liver cirrhosis [33]. Liver stiffness measurement is a non-invasive marker of hepatic fibrosis and has recently been reported to predict the HCC risk [34–36], but the prognostic significance of liver stiffness measurements is yet to be validated in established cirrhosis patients. Our multivariate analysis indicated that liver volume was an independent predictor of HCC regardless of presence of cirrhosis, and sensitivity analysis showed that liver volume index remained as an independent factor in the subgroup of cirrhotic patients. Taken together, we suggest that the additional predictive information of liver volume index may help sub-classify the HCC risk of compensated cirrhosis according to the degree of volume shrinkage.

Several HCC prediction systems have been developed based on traditional risk factors for HCC [27–29]. Liver volume-based nomogram showed fair calibration profiles, and discrimination analysis demonstrated that nomogram-based volume score was able to define high- and low-risk group for future HCC. Moreover, time-dependent ROC analysis revealed that nomogram-based volume score had superior performance compared to HCC prediction models based on conventional predictors. Taken together, our volume-based model may be clinically useful for identifying a “super-high” risk subgroup, i.e. volume score > 150, among CHB patients who received CT scans during surveillance, since these patients may need enhanced follow-up despite negative CT findings for HCC, as described above. Since our cohorts received CT studies for diagnostic rather than prognostic purposes, however, the general application of CT volumetry for HCC prediction needs validation by prospective observations in surveillance cohorts.

For liver volumetry, we used ImageJ freeware with the semi-automatic selection tool to minimize measurement errors. Thanks to the edge-detecting tool, measurement of one patient took approximately 5–10 minutes in experienced hands. This method can be easily implemented in any centre without additional resources as long as digital CT images are available for analysis.

There are several limitations in this study. Firstly, our prediction model was developed and validated in patients who previously received multidetector CT scans and inherently had higher risk for HCC. Therefore, the usefulness of the liver volume index / score cannot be generalized to all CHB population as discussed above. Rather, the main application of volume score is limited to patients for whom multidetector CT scans are already available or requisite by the recall policy during HCC surveillance. Secondly, the retrospective nature of our study warrants further validation by larger prospective design. Thirdly, the validation set was constructed by completed-case analysis [24] and had relatively small samples. Although the validation set gave concordant results with the derivation set and bootstrapping methods were used to increase validity of our results, further validation would be needed. Fourthly, potent oral antiviral agents may regress hepatic fibrosis, but our study was not long enough to observe the potential follow-up changes in liver volumes. Longer follow-up analysis is warranted, preferably with changes in liver volume indices. Finally, liver stiffness data were not available for our patients. Because the fibrosis stage may be related to the liver volume and HCC risk [35, 36], comparison between volumetry and liver stiffness measurement is needed to optimize disease stage-dependent prediction models for HCC.

In conclusion, CT-measured liver volume is an independent predictor of future HCC development, and volume-based prediction model can identify CHB patients with especially high-risk despite negative CT results for HCC during surveillance.

## Supporting information

S1 Table Cox HCC prediction model adjusted for hospital visit frequencies. (DOCX)

S1 Fig Bland-Altman plot showing inter-observer variability of liver volumetry. Differences in measured volumes (%) were plotted against average of liver volumes. The limits of agreement (95% CI) ranged between -7.4% and 5.4%, and 3.3% (3/90) of measurements lay outside the 95% limits of agreement.
(TIF)

S2 Fig Calibration curves of nomogram-based HCC prediction model. The nomogram-predicted 2, 4, and 6-year HCC-free survival rates were plotted against observed HCC-free survival rates at the top, middle a nd bottom row, respectively (left column, derivation cohort; right column, validation cohort). Data were calculated with 100 bootstraps, and error bars indicate 95% confidence intervals.
(TIF)

S1 Video Measurement of liver volume by ImageJ software. (MP4)
