# Supplementary material for: Liver volume-based prediction model stratifies risks for hepatocellular carcinoma in chronic hepatitis B patients on surveillance
Source: PLoS One. 2018 Jan 2;13(1):e0190261. doi: 10.1371/journal.pone.0190261 (PMC5749771; doi:10.1371/journal.pone.0190261)
Supplement: S1 Table — (DOCX) [file pone.0190261.s001.docx]

**S1 Table. Cox HCC prediction model adjusted for hospital visit frequencies**

| Parameters | HR | 95% CI | P value |
| --- | --- | --- | --- |
| Age | 1.04 | 1.01 – 1.06 | 0.008 |
| Male sex | 1.88 | 1.04 – 3.38 | 0.036 |
| Cirrhosis | 4.32 | 1.45 – 12.89 | 0.009 |
| Platelet | 1.00 | 0.99 – 1.00 | 0.235 |
| Hypovascular nodule(s) | 1.11 | 0.65 – 1.91 | 0.707 |
| Volume Index^*^ | 3.88 | 1.62 – 9.31 | 0.002 |
| Frequency of hospital visits (/yr) | 1.44 | 1.28 – 1.62 | < 0.001 |
