# Supplementary figures and images for: Liver volume-based prediction model stratifies risks for hepatocellular carcinoma in chronic hepatitis B patients on surveillance
Source: PLoS One. 2018 Jan 2;13(1):e0190261. doi: 10.1371/journal.pone.0190261 (PMC5749771; doi:10.1371/journal.pone.0190261)

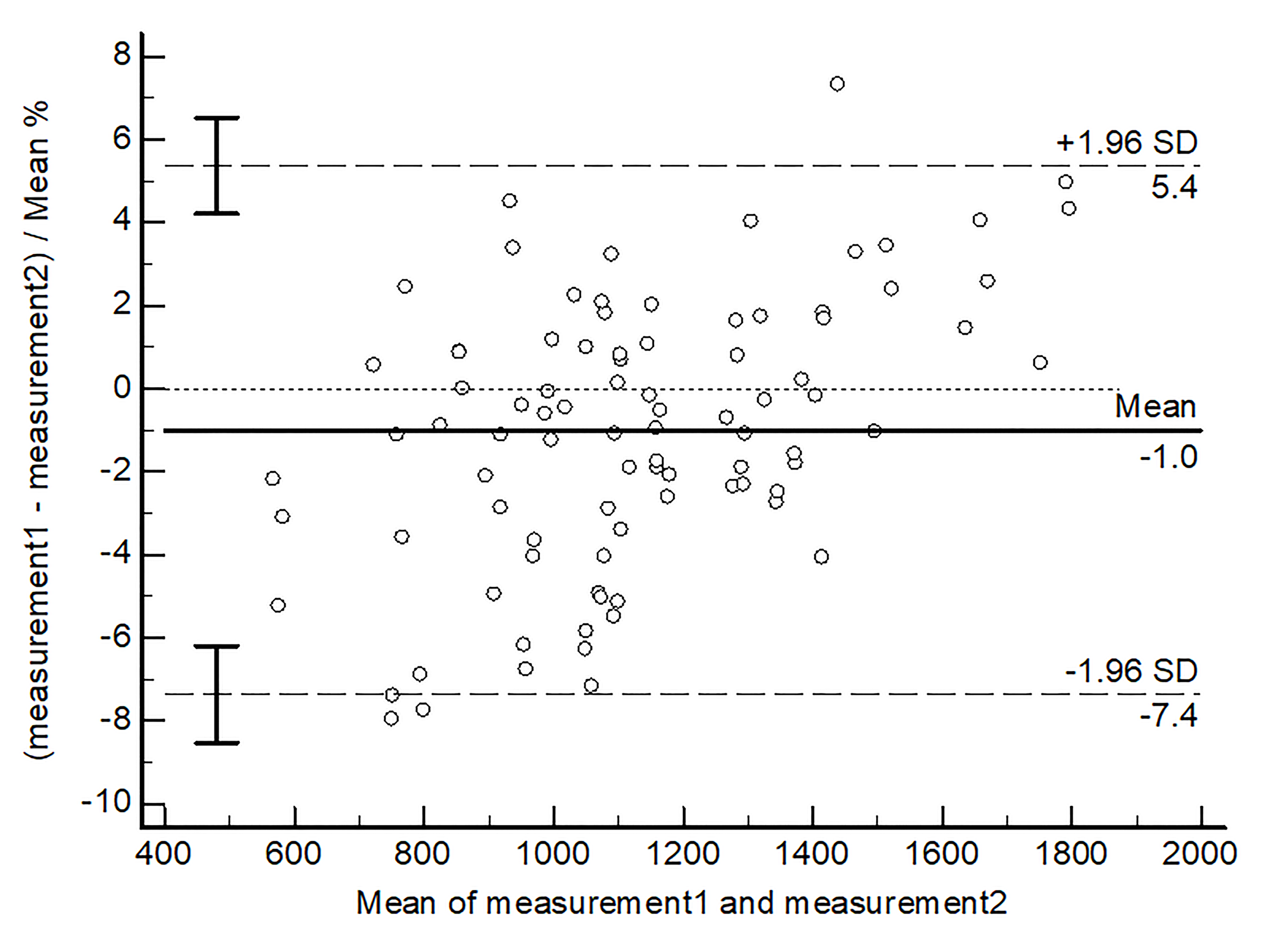

Supplement: S1 Fig — Differences in measured volumes (%) were plotted against average of liver volumes. The limits of agreement (95% CI) ranged between -7.4% and 5.4%, and 3.3% (3/90) of measurements lay outside the 95% limits of agreement. (TIF) [file pone.0190261.s002.tif]

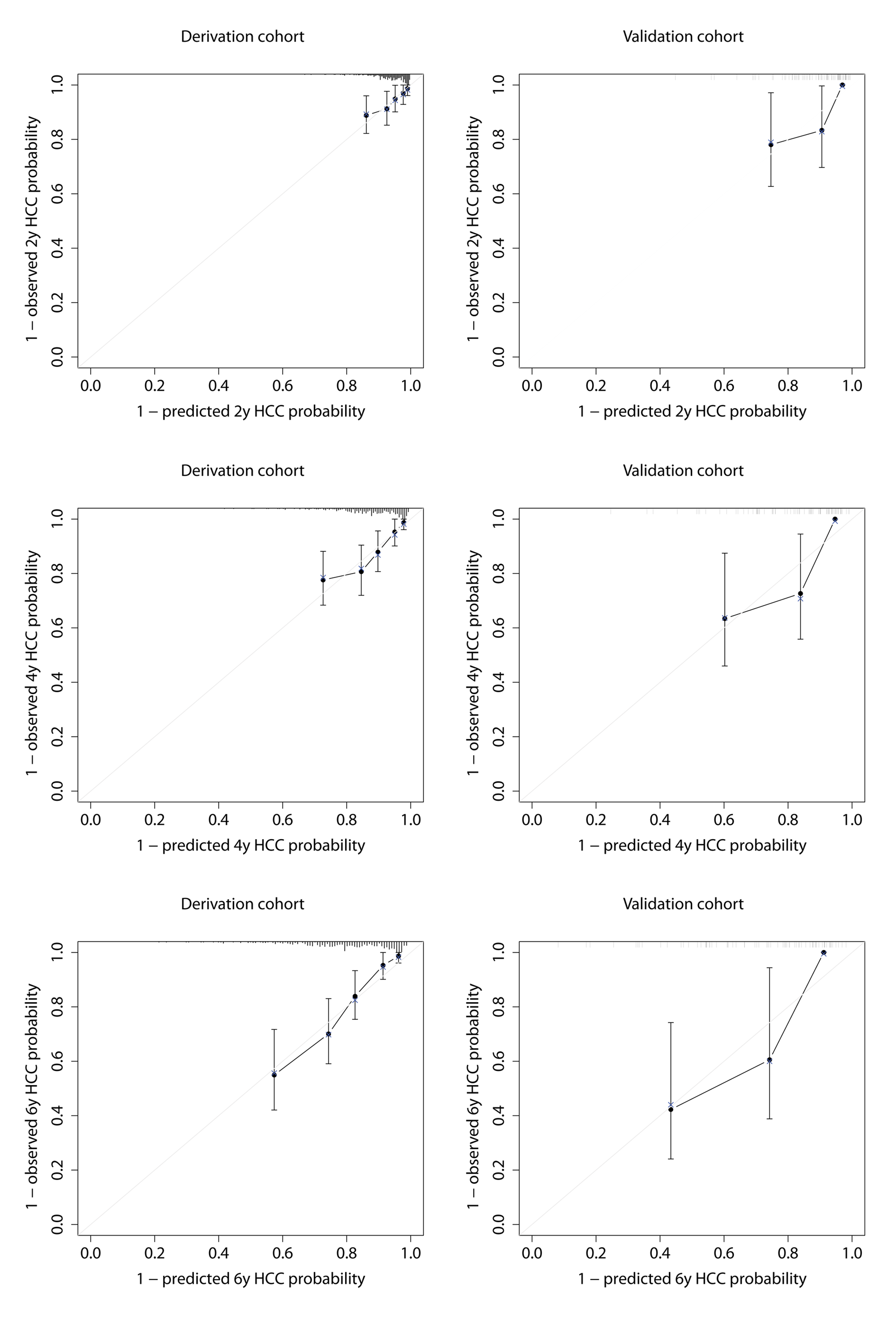

Supplement: S2 Fig — The nomogram-predicted 2, 4, and 6-year HCC-free survival rates were plotted against observed HCC-free survival rates at the top, middle a nd bottom row, respectively (left column, derivation cohort; right column, validation cohort). Data were calculated with 100 bootstraps, and error bars indicate 95% confidence intervals. (TIF) [file pone.0190261.s003.tif]
